# Evaluation of spot urine CA 19 − 9/creatinine ratio in diagnosis and management of hydronephrosis in children: a prospective study

**DOI:** 10.1186/s12887-025-06453-3

**Published:** 2025-12-20

**Authors:** Mehmet Umut Kutukoglu, Turker Altuntas, Cagri Akin Sekerci, Sultan Seval Yilmaz, Goncagul Haklar, Kamil Cam, Tufan Tarcan, Selcuk Yucel

**Affiliations:** 1 Department of Urology, School of Medicine, Marmara University, Fevzi Çakmak Mah., Muhsin Yazicioglu Cad. No:10 Ust Kaynarca/ Pendik, Istanbul, Turkey; 2 Department of Urology, Division of Pediatric Urology, School of Medicine, Marmara University, Istanbul, Turkey; 3 Department of Biochemistry, School of Medicine, Marmara University, Istanbul, Turkey; 4 Department of Urology, School of Medicine, Koç University, Istanbul, Turkey

## Abstract

**Background:**

To demonstrate that the CA19-9/creatinine level could be a potential biomarker in children with obstruction by including ureteropelvic junction obstruction (UPJO) patients and comparing them not only with healthy controls but also with children with non-obstructive hydronephrosis (HN).

**Materials and methods:**

Children with HN and healthy controls with no HN were prospectively enrolled into study from March 2021 to December 2022. The children with HN were divided into two separate groups. Group 1 consisted of children with UPJO, while Group 2 included children with lower urinary tract dysfunction (LUTD). Data collected from study and control groups included age, gender, spot urine CA 19 − 9/Cr ratio at the initial visit and at the 6-month follow-up, the Society of Fetal Urology (SFU) grade, antero-posterior (AP) diameter, renal function and diuretic response on mercaptoacetyltriglycine (MAG-3) dynamic renal scintigraphy and surgical or conservative management selection. Spot urine CA 19 − 9 levels were measured using the ELISA method.

**Results:**

Out of 283 children, Group 1 consisted of 40 children (11 girls, 29 boys; median age: 7 years), Group 2 comprised 18 children (10 girls, 8 boys; median age:8 years) and 225 healthy controls (83 girls, 142 boys; median age: 8 years). The spot urine CA 19 − 9/Cr ratio was significantly higher in the Group 1 and 2 compared to controls (respectively 86.7, 64.5, 47.1 U/mg Cr, *p* = 0.0001). When the two patient groups with HN were compared with each other, no statistically significant difference was observed in the urinary CA19-9/creatinine levels (*p* = 0.358). No correlation was found between CA 19 − 9/Cr ratio and HN severity and/or dynamic renal scintigraphy findings. Surgical management of unilateral UPJO revealed a decrease in CA 19 − 9/Cr in the study group.

**Conclusion:**

This study suggests that the spot urine CA 19 − 9/creatinine ratio may serve as a promising, non-invasive biomarker for distinguishing children with unilateral UPJO and non-obstructive HN from healthy controls. Although elevated levels in patients and postoperative decline were observed, its inability to predict HN severity or the necessity for surgical intervention limits its clinical utility.

Keywords: CA19-9, biomarker, ureteropelvic junction obstruction, hydronephrosis, lower urinary tract dysfunction

## Introduction

Hydronephrosis (HN) is a common congenital urinary system abnormality in all age groups of children. Defining the ureteropelvic junction obstruction (UPJO) is essential to decide the appropriate management to prevent possible kidney damage.

Diagnostic tests should be easy, non-invasive, and cost-effective to perform in detecting the presence of UPJO and also in predicting the severity of the condition. Another goal of those tests is to decide who would benefit from the early surgical treatment to preserve the renal function [1]. Although imaging studies with different modalities is the backbone of diagnostic work-up in differential diagnosis of HN, there is no easy way to distinguish the best candidates for surgery to relieve UPJO [2]. Given the complexity of the association of HN and UPJO, there is a substantial need for biomarkers to aid in predicting the severity of UPJO and deciding on surgical interventions and also, evaluating the success of treatments [3]. There have been some studies on possible urinary biomarkers to identify the presence and severity of UPJO and to predict the severity of UPJO and function of the effected kidney [4].

Carbohydrate antigen 19 − 9 (CA 19 − 9) is a carbohydrate antigen associated with tumors, originally identified using a monoclonal antibody against a human colorectal cancer cell line. It is commonly used as a tumor marker for pancreatic and gastrointestinal conditions, including those affecting the gallbladder, pancreas, stomach, colon, bronchial system, endometrium, salivary glands, and prostate [5]. CA 19 − 9 is a possible urinary biomarker since its expression from renal pelvis epithelium was first reported by Ohshio and colleagues [6]. Although it is basically secreted from pancreas, stomach, colon, bronchial tree, endometrium, salivary glands and prostate secretions [7], its association with renal pathologies particularly HN has been studied with diverse results [5, 8–12].

As mentioned above, there are many studies in the literature investigating the relationship between CA19-9 and HN [13–15]. However, upon reviewing these studies, we observed that patients with UPJO are generally compared only with a healthy control group. In our prospective study, we aimed to demonstrate that the CA19-9/creatinine level could be a potential biomarker in children with obstruction by including the largest sample size of UPJO patients and comparing them not only with healthy controls but also with children with non-obstructive HN.

## Materials and methods

All children aged between 0 and 16 years-old were included in this prospective study at our pediatric urology clinic between October 2021 and December 2022. The study was approved by the Ethics Committee of University (Approval Number: 09.2021.167). Informed consent was obtained from the parents of all participants before participation.

The children included in the study were divided into three groups for evaluation. The Group 1 consisted of children with unilateral HN due to UPJO, the Group 2 included children with unilateral low-grade HN due to lower urinary tract symptoms (LUTD), and the third group consisted of healthy children as the control group. Of the patients with LUTD in Group 2, 12 had overactive bladder and 6 had dysfunctional voiding.

Since we aimed to homogenize the study group, children with bilateral HN, prior urinary system surgery, urinary stone disease, solitary kidney, active urinary tract infection (UTI), diabetes, malignancy, chronic kidney failure, not complying with imaging tests and failed to follow-up were excluded from the study.

All children who were diagnosed with unilateral HN at initial urinary ultrasonography were reevaluated for standardized SFU grading, AP diameter and underwent diuretic dynamic renal scintigraphy with technetium-99 m mercaptoacetyltriglycine (99 m Tc-MAG3). As recommended by the EAU Guidelines [16], MAG3 scintigraphy considered the gold standard, especially in the pediatric population, for evaluating the severity of UPJ obstruction and split renal function was used. This is one of the most important studies employed to determine the split renal function of each kidney and to identify any evidence of renal obstruction in children with HN detected on ultrasound, after excluding other possible causes of HN such as vesicoureteral reflux, and urinary system stones. According to the results of MAG3 scintigraphy, patients with HN were divided into 2 groups as obstructed and non-obstructed.

Surgical management for UPJO was selected in children according to EAU Guidelines with impaired split renal function (< 40%), a decrease of split renal function of > 10% in subsequent studies, poor drainage function after the administration of furosemide, increased anteroposterior diameter on US, and grade III and IV dilatation as defined by the SFU [17].

In this study, 23 patients in Group 1 underwent dismembered pyeloplasty using the Anderson-Hynes technique. Of these, 20 were non-responders to diuretic challenge on MAG3 scintigraphy, while the remaining 3 patients, despite showing a diuretic response, underwent surgery due to the presence of pain and deterioration in renal function during follow-up.

Age, gender, and spot urine CA 19 − 9/Creatinine measurements at first visit and at 6th month visit following conservative or surgical treatment were recorded in both arms.

Spot urine samples were collected from all patients under standardized conditions. Samples were obtained between 9:00 and 10:00 a.m., after at least 3 h of fasting, and patients were instructed to maintain normal hydration. This procedure was applied consistently to all patients to minimize potential variations due to diurnal rhythm, food intake, or hydration status.

Urine samples for CA 19 − 9 analysis were centrifuged at 3500 rpm for 4 min, and 15 mL of the supernatant was stored at −80 °C for further analysis. Spot urine CA 19 − 9 levels were measured using the ELISA method with the BT LAB ELISA kit on the DAR800 ELISA analyzer, following the manufacturer’s protocol.

Firstly, the demographic data as well as the baseline and 6th-month CA19-9/creatinine levels of the Group 1, Group 2, and the healthy control group were compared. Subsequently, group 1 were subdivided based on whether they underwent surgery and according to their diuretic response on MAG3 scintigraphy, and separate analyses were performed for these subgroups. Afterwards, correlation analyses were conducted between CA19-9/creatinine levels and various parameters in both the control group and the group 1. Finally, a ROC analysis was performed in the group 1 to determine the cut-off value of the CA19-9/creatinine ratio for the presence of HN.

### Statistical analysis

Statistical analyses were conducted using IBM SPSS version 25.0. The Kruskal Wallis test assessed data normality. For numeric data, the Mann-Whitney U and Kruskal-Wallis tests were used for independent groups, and the Wilcoxon test for dependent groups. Chi-square test was used for categorical parameters. Spearman correlation analysis was performed to evaluate the relationship between two non-parametric variables. ROC analysis was used to determine the threshold for the best sensitivity and specificity of spot urine CA 19 − 9/Cr levels.

The G-power program was used for power analysis. Study by Alizadeh et al., which investigated CA 19− 9 as a potential biomarker in patients with UPJO, was used as a reference [5]. A total of 62 participants (31 patients, 31 controls) were required for 95% power (α = 0.05) based on a standard deviation of 1.18 U/mg cre and an expected 1 U/mg cre difference in CA19-9/Cr levels.

## Results

A total of 283 children were enrolled in this prospective study. 40 children with unilateral UPJO and 18 children with low grade HN and LUTD were in the study group and 225 healthy children were in the control group. At the time of first visit, median spot urinary CA19-9/creatinine values were compared among the three groups, a statistically significant difference was observed between Group 1 (86.7) and the control group (47.1), as well as between Group 2 (64.5) and the control group (*p* < 0.001 and *p* = 0.009, respectively). However, no significant difference was found between Group 1 and Group 2 in terms of CA19-9/creatinine values (*p* = 0.358) (Table 1).

**Table 1:** Comparison of parameters measured in spot urine at first presentation among children

	Control Group (*n* = 225)	Group 1 (*n* = 40)	Group 2 (*n* = 18)	*P* value
Age (years)				
Median (min-max)	8 (1-16)	7 (1-15)	8 (5-16)	**0.044**
Sex				
(Female/Male) (%)	83/142 (36.9/63.1)	11/29 (27.5/72.5)	10/8 (55.6/44.4)	0.122
First visit CA 19-9 (U/mg Cr)				
Median (min-max)	47.1 (14.1-165)	86.7 (28.3-243)	64.5 (38.2-142)	**0.001**
6^th^ Month CA 19-9 (U/mg Cr)				
Median (min-max)	44.6 (14-140)	59.2 (22.5-177.7)	55.8 (28.2-115.8)	**0.001** ^**#**^

(*CA 19-9 *Carbohydrate antigen 19-9, *U/mg Cr *Units per milligram creatinine), (#=control vs. Group 1) Values in bold represent statistically significant differences (p < 0.05)

When the Group 1 was analyzed, the median CA19-9/creatinine level was calculated as 92.2 in the surgically treated group and 80.1 in the conservatively managed group. Although the median value was higher in the surgical group, no statistically significant difference was observed between the two subgroups. In the study group of 23 patients who underwent surgery for unilateral UPJO, the initial spot urine CA 19 − 9/Cr ratio was 92.2 (28.3–243), which decreased to 53 (22.5–143.4.5.4) U/mg Cr at 6 months postoperatively. A statistically significant decrease in CA19-9/creatinine levels was observed in this patient group after surgery (*p* = 0.001). Finally, when a subgroup analysis was performed in the Group 1 according to the diuretic response on MAG3 scintigraphy, the median CA19-9/creatinine level was calculated as 80 in the diuretic-responsive group and 102.1 in the non-responsive group. However, no statistically significant difference was found between the two subgroups (Table 2).

**Table 2:** Subgroup analysis in Group 1

Parameters		*n* (%)	CA 19-9/Creatinine (U/mg Cr) Median (range)	*p*
First Visit	Conservative group	17 (42.5%)	80.1 (40.7-211.6)	*p *= 0.420
	Surgery group	23 (57.5%)	92.2 (28.3-243)	
Surgery group	First Visit	23 (100%)	92.2 (28.3-243)	***p*** **= 0.001**
	Postoperative 6^th^ Month	23 (100%)	53 (22.5-143.4)	
MAG-3	Diuretic Responsive	20 (50%)	80 (46.6-211.6)	*p *= 0.372
	Diuretic Unresponsive	20 (50%)	102.1 (28.3-243)	

(*CA 19-9*: Carbohydrate antigen 19-9, *U/mg Cr* Units per milligram creatinine, *Range* Minimum- maximum, *MAG-3 *Mercaptoacetyltriglycine)Values in bold represent statistically significant differences (p < 0.05)

Subsequently, when a correlation analysis was performed in the control group, a positive correlation was also detected between the baseline CA19-9 level and the 6th-month CA19-9 level. However, no correlation was found between CA19-9 levels and age in the control group (Table 3).

**Table 3:** Correlation analysis of spot urine CA 19-9/Creatinine levels for control group

	CA 19-9 /Creatinine	6. month CA 19-9 /Creatinine	Age
CA 19-9 /Creatinine	1.000	.886^**^	-0.114
6. month CA 19-9 /Creatinine	.886^**^	1.000	-0.077
Age	-0.114	-0.077	1.000

(*CA 19-9 *Carbohydrate antigen 19-9)

(Spearman Correlation Analyses *p<0.05, **p<0,01)

The results of the correlation analysis indicated in the Group 1 between the postoperative 6th-month CA19-9 level, AP diameter, SFU grade, and split function values obtained from MAG3, a statistically significant positive correlation was found between the baseline CA19-9 level and the postoperative 6th-month CA19-9 level. In addition, a similarly significant correlation was found between the AP diameter and SFU grade (Table 4).

**Table 4:** Correlation analysis of spot urine CA 19-9/Creatinine levels for group 1

	CA 19-9 /Creatinine	PO 6. month CA 19-9 /Creatinine	AP Diameter	SFUGrade	MAG-3 Function
CA 19-9 /Creatinine	1.000	.762^**^	-0.044	0.186	-0.036
PO 6. month CA 19-9 /Creatinine	.762^**^	1.000	-0.228	-0.100	0.210
AP Diameter	-0.044	-0.228	1.000	.646^**^	-0.177
SFUGrade	0.186	-0.100	.646^**^	1.000	-0.228
MAG-3 Function	-0.036	0.210	-0.177	-0.228	1.000

(*CA 19-9 *Carbohydrate antigen 19-9, *PO* Post operative, *AP* Antero-posterior, *SFU* Society of Fetal Urology, *MAG-3* Mercaptoacetyltriglycine)

(Spearman Correlation Analyses *p < 0.05, **p < 0,01)

**Fig. 1:**
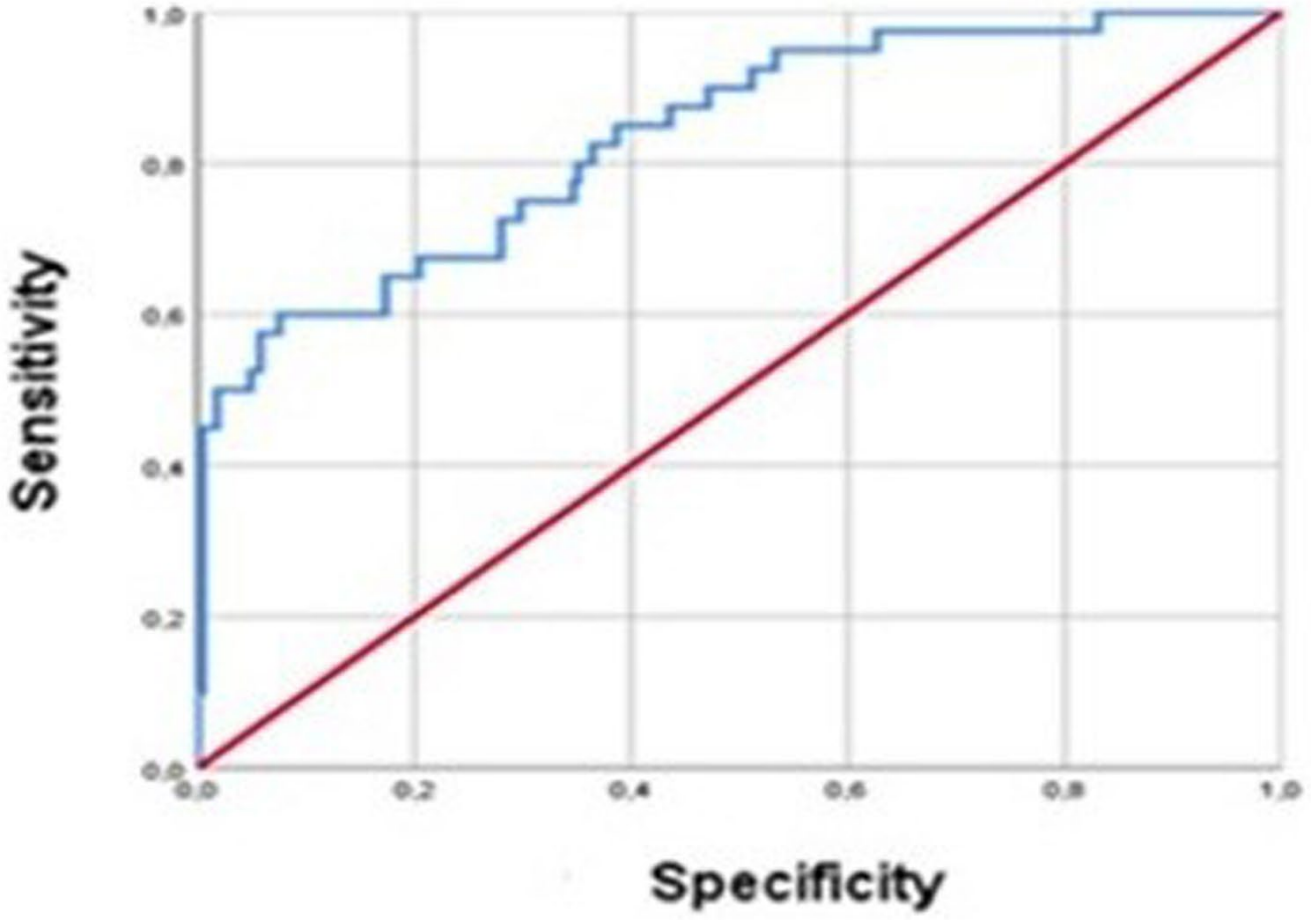
ROC analysis of spot urine CA 19-9/Cr in detecting hydronephrosis

In our study, considering the presence of HN, the ROC analysis yielded an area under the curve (AUC) of 0.798 (95% confidence interval 0.734–0.863) with sensitivity of 69% and specificity of 68.9%. The threshold value for spot urine CA 19 − 9/Cr was determined as 57 U/mg Cr (*p* = 0.0001) (Fig. 1).

## Discussion

In children, HN is a common radiological urinary system finding requiring further invasive and non-invasive tests to distinguish the severity of disorders and identify the obstructing cause and to monitor the progress of renal function during conservative and surgical treatment. In the control group, spot urine CA 19 − 9/Cr (U/mg) values showed no significant correlation with age (*p* = 0.105) and did not differ significantly between sexes (*p* = 0.33). These findings suggest that the spot urine CA 19 − 9/Cr ratio is a more stable biomarker across both sexes and all pediatric age groups, although these data are not included in the results section. The spot urine CA 19 − 9/Cr ratio effectively distinguished the unilateral UPJO from healthy children (*p* = 0.0001) (Table-1). Nearly half of the patients in Group 1 had SFU grade 1 HN; these cases may represent mild or clinically insignificant obstruction. This could potentially reduce the differences in biomarker levels between groups, thereby affecting the strength of the observed associations. We believe that this should be taken into consideration. Following conservative and surgical treatment in UPJO patients, the resolution of HN was compatible with a decrease in the spot urine CA 19 − 9/Cr ratio (*p* = 0.001) (Table 2). All children with surgical management revealed resolution of HN and no complications or failures occurred.

Mechanical strain on tubular cells associated with HN leads to apoptosis, inflammation, interstitial fibrosis and oxidative stress, ultimately resulting in nephron loss and potentially leading to kidney failure [18]. It was postulated that CA 19 − 9 expressed in the apical cells of the collecting system epithelium is shed into urine in response to mechanical strain, leading to its detection in high amounts in urine [6]. Therefore, CA 19 − 9 was thought to be a possible biomarker to detect HN. Although there have been studies on the use of spot urine CA 19 − 9 values to predict the HN [8–11, 19]., the spot urine CA 19 − 9/Cr (U/mg) ratio has been promoted only in three studies [5, 12, 20].

As seen in Table 1, we showed that CA 19 − 9/Cr ratio in spot urine efficiently detects unilateral UPJO in children. Alizadeh et al. also found that the spot urine CA 19− 9/Cr ratio was a more accurate indicator in obstruction cases (*p* = 0.0001) [5]. Similarly, Miranda et al. reported that CA 19− 9/Cr ratio in spot urine was the best biomarker among KIM-1, NGAL, CA 19 − 9 and Beta-2 Microglobulin to detect HN related to UPJO in 47 patients compared to 40 controls in adults [20]. They stated that 51.3 U/mg Cr value would have 48.9% sensitivity and 88% specificity for HN related to UPJO. We found that 57 U/mg Cr value of CA 19 − 9/Cr for a sensitivity of 69% and specificity of 68.9% for HN as seen in Fig. 1 (*p* = 0.0001). Differences in threshold values and diagnostic performance may be attributed to several factors, including patient selection, the proportion of mild versus severe HN cases, variations in urine collection protocols, and the timing of sample acquisition. For instance, our cohort included a substantial number of SFU grade 1 HN cases, which may have increased the threshold required to achieve comparable sensitivity. Additionally, the fact that the referenced study was conducted in adults while our study was performed in children may also contribute to these differences. These findings emphasize the importance of considering study-specific factors when interpreting biomarker thresholds and suggest that the CA 19 − 9/Creatinine ratio should be validated in larger, standardized cohorts before clinical implementation.

All studies [5, 8–12, 20] but one by Amini et al. [19] focused on only UPJO as the underlying pathology for HN. In Kajbafzadeh et al.’s study involving 27 UPJO patients and 41 controls, they observed a significant correlation between AP diameter and urinary CA 19 − 9 levels [10]. However, Atar et al. found no significant correlation between spot urine CA 19− 9 levels and prenatal or third-trimester AP diameters in UPJO patients [8], and Amini et al. also reported no significant relationship between spot urine CA 19− 9 levels and HN severity [19]. However, we found that there is a tendency to have a positive correlation between the severity of HN and CA 19 − 9/Cr ratio, although it is not statistically significant.

Another strong finding in our study is the association of a decrease in CA 19 − 9/Cr ratio level with resolution of HN. As seen in Table 2, surgical treatment of unilateral UPJO leading to HN resolution is accompanied by a significant decrease in CA 19 − 9/Cr ratio in spot urine (*p* = 0.001). Our results are also supported by other authors’ studies. Atar et al. studied patients undergoing pyeloplasty for UPJO and found that spot urine CA 19− 9 levels significantly decreased by three months post-surgery. They also observed no correlation between CA 19 − 9 levels and age in both patient and control groups, like our findings [8].

Some studies advocated the potential use of urinary CA 19 − 9 to define the best candidates for UPJO surgical treatment. A higher urinary CA 19 − 9 level or ratio to urinary Cr was claimed to be associated with diuretic nonresponse in dynamic renal scintigraphies and/or decreased renal function on the effected renal unit. Nabavizadeh et al. studied 112 patients diagnosed with UPJO, categorizing them into groups based on their management indications: closely monitored, requiring pyeloplasty during follow-up, and requiring pyeloplasty at diagnosis. They found statistically significant differences in spot urine CA 19 − 9 levels among these groups measured at diagnosis as highest CA 19 − 9 level with the surgical indication requirement [11]. Özkuvancı et al. separated their study population into obstructive UPJO, non-obstructive UPJO, and healthy controls. Median spot urine CA 19− 9/Cr ratios differed significantly among those groups [12] (*p* < 0.001). As seen in Table 2, in our prospective study, we failed to show such correlation between urinary CA 19 − 9/Cr ratio and radiologic indications for surgical treatment of UPJO such as HN severity, diuretic response or renal function. However, we should note that we used urine from urinary bladder where Özkuvancı et al. from the renal pelvis during surgical treatment. While CA 19− 9/Cr levels showed a postoperative decrease in our cohort suggesting that the marker may reflect dynamic changes after relief of obstruction its lack of predictive value for disease severity or surgical necessity limits its clinical applicability at this stage. Therefore, although CA 19 − 9 remains a promising non-invasive urinary biomarker, current evidence indicates that it is not yet ready for routine clinical decision-making, and further studies with standardized sampling and larger subgroups are needed.

Another interesting finding in our study was the children in Group 2. As seen in Table 1, CA 19 − 9/Cr value was high compared to control groups 64.5 (38.2–142), *p* = 0.001). As illustrated in Table 1, children in Group 2 undergo urotherapy with/without antimuscarinics and CA 19 − 9/Cr ratio decreases levels with resolution of HN (64.5 (38.2–142) to 55.8 (28.2–115.8.2.8). However, non-obstructed HN failed to show a difference in CA 19 − 9/Cr ratio level after 6 months of follow up with appropriate management with/without antimuscarinic medication. In the systematic review of Şekerci et al., it is observed that many biomarkers are used in the evaluation of these patient groups [21]. However, we would like to note that CA 19 − 9 was evaluated for the first time in our study of non-obstructed HN patients.

It is important to note the limitations of our study. A major limitation of this study is the relatively small sample size in both the non-obstructive HN group and the surgical subgroup, which may limit the statistical power and the generalizability of the findings. Future studies with larger cohorts are needed to validate these results. SFU grade 1 HN cases compose nearly half of the study group. Due to the low number of patients in the Group 1, the strength of evidence regarding the CA 19 − 9/Creatinine ratio in treatment decision-making is limited. However, the overall number of children in the HN and control groups is high, and the method of measuring the CA 19 − 9/Creatinine ratio is reliable. However, each case is reevaluated and retested in our clinic for standardized reporting. Finally, we believe that more frequent CA 19 − 9/Cr measurements during postoperative follow-up could have strengthened our evaluation. The lack of a 3-month follow-up measurement represents a limitation of our study.

## Conclusion

Our study highlights the potential of the spot urine CA 19 − 9/creatinine ratio as a promising, non-invasive biomarker for differentiating children with unilateral UPJO and non-obstructive HN from healthy controls. Considering these findings, although this biomarker was found to be significantly elevated in the patient group compared to healthy controls and showed a decrease after surgery, its inability to reflect the severity of HN or the need for surgical intervention raises some concerns.

## Data Availability

Data are however available from the authors upon reasonable request and with permission.
